# The Influence of pH, NaCl, and the Deacidifying Yeasts *Debaryomyces hansenii* and *Kluyveromyces marxianus* on the Production of Pigments by the Cheese-Ripening Bacteria *Arthrobacter arilaitensis*

**DOI:** 10.3390/foods7110190

**Published:** 2018-11-19

**Authors:** Nuthathai Sutthiwong, Mireille Fouillaud, Laurent Dufossé

**Affiliations:** 1 The Expert Centre of Innovative Health Food (InnoFood), Thailand Institute of Scientific and Technological Research, Technopolis 35 Mu 3, Klong Ha, Klong Luang, Pathum Thani 12120, Thailand; nuthathai@tistr.or.th; 2 Laboratoire de Chimie des Substances Naturelles et des Sciences des Aliments (LCSNSA), Université de La Réunion, ESIROI Département Agroalimentaire, Parc Technologique, 2 rue Joseph Wetzell, F-97490 Sainte-Clotilde, Ile de La Réunion, France; mireille.fouillaud@univ-reunion.fr

**Keywords:** *Arthrobacter arilaitensis*, coloration, pigmentation, ripened cheese, spectrocolorimetry

## Abstract

*Arthrobacter arilaitensis* is a food-related bacterial species under investigation for its involvement in the coloration of surface-ripened cheeses. Presently, information about this species in association with the development of appropriate cheese coloration is still lacking. This study was performed in order to investigate—with the use of spectrocolorimetry—the influence of pH, NaCl, and deacidifying yeasts on the pigmentation of *Arthrobacter arilaitensis* biofilms. Three types of cheese-based (curd) solid media were prepared by using different deacidification methods: (i) chemical deacidification by NaOH (CM_NaOH_); (ii) biological deacidification by the yeast strain *Debaryomyces hansenii* 304 (CM_Dh304_); and (iii) biological deacidification by the yeast strain *Kluyveromyces marxianus* 44 (CM_Km44_). Each medium was prepared with initial pH values of 5.8, 7.0, and 7.5. After pasteurization, agar was incorporated and NaCl was added in varying concentrations (0%, 2%, 4%, and 8% (w/v)). *A. arilaitensis* Po102 was then inoculated on the so prepared “solid-curd” media, and incubated at 12 °C under light conditions for 28 days. According to the data obtained by spectrocolorimetry in the Compagnie Internationale de l’Eclairage (CIE) L*a*b* color system, all controlled factors appeared to affect the pigments produced by the *A. arilaitensis* strain. NaCl content in the media showed distinct inhibitory effects on the development of color by this strain when the initial pH was at 5.8. By contrast, when the initial pH of the media was higher (7.0, 7.5), only the highest concentration of NaCl (8%) had this effect, while the coloring capacity of this bacterial species was always higher when *D. hansenii* 304 was used for deacidification compared to *K. marxianus* 44.

## 1. Introduction

The color of smear-ripened cheese rind is one of the major attributes determining a consumer’s purchasing decision, and it is linked with several cheese qualities, e.g., maturity, flavor, and cleanliness [1]. A large number of smear-ripened cheeses are recognized by consumers through their rind’s characteristics, especially the presence of color and flavor, which depend on the relevant properties of each cheese. The surface of smear-ripened cheeses is the result of pigment synthesis by a complex surface microflora, consisting of various species, both prokaryotes and eukaryotes [2]. Due to its presence at different stages of the ripening process, the genus *Arthrobacter* has long been identified in smear-ripened cheeses as one of the important bacteria among a diversity of microorganisms [3,4,5,6,7]. One dominant strain of this genus that was found in cheeses is *Arthrobacter arilaitensis* (presently named *Glutamicibacter arilaitensis*—taxonomy always evolves and we choose to keep the name *Arthrobacter arilaitensis* in this publication, in agreement with our previous articles on color and dairy), which is present during ripening, particularly at the middle and late stages; thus, it can be assumed that *A. arilaitensis*, whose colonies commonly exhibit yellow color, could be one of the microorganisms that is significantly responsible for the pigmentation on cheese rind, contributing to its characteristic overall color [8,9]. Recently, yellow pigments produced by cheese-ripening *A. arilaitensis* strains were characterized as comprising eight different C50 carotenoids, mainly decaprenoxanthin [10,11].

During the cheese-making process, the ripening creates complex microbial communities, which vary in terms of both microorganisms’ abundance and diversity. This step is essential as the formulation of smear determines some major organoleptic properties of the cheese, such as color, flavor, and texture [6,12]. Surface ripening begins with the growth of yeasts, depending on the cheese variety, mainly *Debaryomyces hansenii*, *Kluyveromyces marxianus*, *Yarrowia lipolytica*, and the yeast-like mold *Geotrichum candidum*, which colonize the cheese surface and metabolize lactic acid during the initial period of ripening [13,14,15]. This metabolism induces the increase of the pH of the cheese’s surface, resulting in the promotion of the growth of acid-sensitive bacteria. Consequently, the development of bacteria on the cheese surface appears to be dependent on the metabolism of lactic acid by yeasts [15,16]. Deacidifying yeasts have been reported to affect both the growth and the pigmentation of smear-ripening bacteria.

According to Leclercq-Perlat et al. [17], the color intensity of pigments produced by *Brevibacterium linens*, which is part of microorganisms found at the surface of smear-ripened cheeses, was higher when using *D. hansenii* for deacidification, than *K. marxianus*. In addition, significant differences of the color intensity provided by ripening bacteria, in combination with different strains of *D. hansenii*, have been demonstrated [18]. However, besides the yeast strains used in the deacidification of the cheeses, other factors, such as light, pH, temperature, and salt concentration, can also influence the pigmentation of cheese-ripening bacteria [19,20,21,22].

Sutthiwong et al. [19] identified three groups of *A. arilaitensis* strains that had different coloration behavior when subjected to light. Some *A. arilaitensis* strains reacted as positively sensitive (i.e., displayed brighter pigmentation under daylight condition than in the darkness), others as negatively sensitive, and some were not sensitive to light. The significant effects of pH and sodium chloride concentration on the intensity of pigments produced by *B. linens* and *Corynebacterium flavescens* were addressed by Masoud and Jakobsen [21]. The same investigation showed that the strongest color intensity was present at pH 7.0, contrasting with pH 6.0 and pH 5.6; meanwhile, the addition of 4% (w/v) NaCl resulted in the highest intensity of the orange-reddish color when compared to 0 and 8%.

To date, the color development by the surface microflora of ripened cheeses is still of great interest to cheese makers, particularly in the production of artisanal cheeses. This then stimulated a large volume of research projects on cheese coloration under the influences of various factors, e.g., interactions between the microorganisms in the smear and physical and chemical factors of the environment for microbial growth. Nevertheless, it seems that the coloration by *A. arilaitensis* was not often investigated, especially its pigmentation as affected by various factors, in contrast to the orange *B. linens* strains. The present study has been performed in order to investigate the effects of three factors in combination, i.e., initial pH, addition of NaCl, and deacidifying yeasts, on the pigmentation by *A. arilaitensis*, using quantitative spectrocolorimetry. 

## 2. Materials and Methods

### 2.1. Strains and Cultures

#### 2.1.1. Yeast and Bacterial Strains

The bacterial and yeast strains used in this study, namely, *Arthrobacter arilaitensis* Po102, *Debaryomyces hansenii* 304, and *Kluyveromyces marxianus* 44, were kindly provided by the Institut National de la Recherche Agronomique (INRA), France. They were maintained during this study on medium depending on their nutrient requirements (i.e., milk ingredient-based medium for bacteria or potato dextrose agar for yeasts, as described below), stored at 4 °C, and subcultured monthly. 

#### 2.1.2. Cultures

Potato dextrose broth (BD Biosciences, Le Pont de Claix, France) was used as a subculture medium for yeasts while the milk ingredient-based medium was prepared and used for bacteria. A milk ingredient-based liquid medium containing 5 g casamino acids (BD Biosciences, France), 1 g yeast extract (BD Biosciences, France), 5 g NaCl (Fisher Scientific, Illkirch-Graffenstaden, France), 20 g D-glucose (Fisher Scientific, France), and 1 g KH_2_PO_4_ (Fisher Scientific, France), per liter of deionized water, was prepared. Before sterilizing at 121 °C for 15 min, the pH of the medium was adjusted to 7.0 ± 0.2.

To prepare a microbial suspension, each of *D. hansenii* 304 and *K. marxianus* 44 was inoculated into 250 mL Erlenmeyer flask containing 50 mL of PDB, whilst the milk ingredient-based liquid medium was used instead of PDB for *A. arilaitensis* Po102. These flasks were incubated at 25 °C with agitation at 150 rpm for 2 days. After the cultivation period, the cells were harvested by centrifugation at 6000× *g* for 10 min, and resuspended in peptone saline diluent (1 g casein peptone (Sigma-Aldrich, Darmstadt, Germany) and 8.5 g NaCl, pH adjusted to 7.0 ± 0.2 at 25 °C)). A suspension containing 10^7^ cells mL^−1^ was prepared to be used in the next step.

### 2.2. Deacidified Cheese-Based Media and Factors Controlled

The procedure for preparing the experimental media employed in this study was adapted according to Leclercq-Perlat et al. [17], Rea et al. [23], and Masoud and Jakobsen [21]. The cheese-based (curd) media were prepared in two steps: (i) the pH adjustment by chemical or biological deacidification, depending on the observation of coloration influenced by yeasts; and (ii) the inoculation of bacterial strains on the media surface.

The cheese curd used throughout the experiment was kindly supplied by Fromagerie de Bourbon, Reunion Island, France. After receiving it, the cheese curd was frozen at −80 °C to prevent enzymatic reaction [24,25].

Before preparing the cheese-based media, the frozen cheese curd was thawed at 4 °C for roughly 24 h. The defrosted cheese curd was mixed with distilled water (60% (w/v)); then the mixture was homogenized using a blender and placed in a water bath (100 °C) for 30 min. After cooling down to 60 °C, the mixture was blended, again, until the texture was homogeneous. This cheese slurry was divided depending on the volume required for each treatment, and then poured into sterilized flasks for further steps.

#### 2.2.1. pH Adjustment

##### Chemical Deacidification

A flask containing cheese slurry was pH adjusted to 5.8, 7.0, or 7.5, with 1 M NaOH (Fisher Scientific), followed by the addition of 2 g granulated agar (BD Biosciences, France) and NaCl (0%, 2%, 4%, or 8% (w/v)). The final mixture was pasteurized by heating at 80 °C for 10 min. After cooling down to 45–50 °C, the pour plate technique was applied to prepare the cheese-based (curd) solid medium contained in Petri dishes, which were left to solidify for 2 h before inoculation with *A. arilaitensis* Po102.

##### Biological Deacidification

When the temperature of the cheese slurry decreased to approximately 30 °C, it was inoculated with a suspension containing 10^7^ cells mL^−1^ of either *D. hansenii* 304 or *K. Marxianus* 44, with a suspension-to-slurry ratio of 1:100 (v/v). After the inoculation, the slurry was homogenized for a while, and then the flask was closed with a cork cap, wrapped in an aluminum foil, and incubated in the darkness at 25 °C while agitating at 150 rpm until the pH values increased to 5.8, 7.0, or 7.5. In order to stop the yeast from affecting the pH value, the cheese slurry was heated at 80 °C for 10 min. Agar with varying NaCl contents, as described before, was added to the mixture. Afterwards, the same procedures as mentioned above in “Chemical deacidification” (after adjusting the pH by a chemical reagent) were applied.

#### 2.2.2. Inoculation of *Arthrobacter arilaitensis* and Cultivation Conditions

After solidifying, one mL of a 10^7^ cell suspension of *A. arilaitensis* Po102 was spread over the surface of the deacidified media. All Petri dishes were then incubated at 12 °C under daylight fluorescent illumination. Three independent replicates for each treatment were collected at 7, 14, 21, and 28 days, and measured using spectrocolorimetry for the values of color coordinates.

### 2.3. Color Measurements

The cheese-based (curd) solid medium inoculated with *A. arilaitensis* Po102 was measured by using a CM-3500d spectrocolorimeter (Minolta Co., Ltd., Hino-shi, Tokyo, Japan) driven with SpectraMagic *NX* Pro. Software (Minolta). The reference illuminant was D65 (standard daylight). According to the Compagnie Internationale de l’Eclairage (CIE, 1978) the data were reported in the L*a*b* colorimetric system. A single-pieced disk of agar, large enough to cover the entire light spot, was cut in each Petri dish, from an area of homogeneous colony development. The sample was then held culture-down, and layered in the bottom of a 45 mm diameter glass Petri dish (CM-A128, Konica Minolta, Hino-shi, Tokyo, Japan) for incident light color measurements.

To characterize a color in the CIE L*a*b* color system, 3 colorimetric coordinates are obtained from the spectrocolorimeter. L* defines lightness (ranges from 0% to 100%, dark to light), a* indicates the red/green value (from −60 to 60, green to red), and b* denotes the blue/yellow value (from −60 to 60, blue to yellow). The attributes of color, C* and *h*°, describe the chroma (vividness or dullness) and the hue angle or tone of the color, respectively. The value of chroma C* is 0 at the center, and increases according to the distance from the center. Hue angle *h*° is defined as starting at the +a* axis and is expressed in degrees: 0° would be +a* (red), 90° would be +b* (yellow), 180° would be −a* (green), and 270° would be −b* (blue). Hue values correspond to the angle of the a*/b* coordinated of the points.

Chroma (C*) and hue angle (*h*°) can be calculated as follows:

$$\mathrm{Chroma}\;C*=\sqrt{{\left(a*\right)}^{2}+{\left(b*\right)}^{2}},$$

$$\mathrm{Hue}\;\mathrm{angle}\;h{}^{\circ}=\tan^{-1}\left(\frac{b*}{a*}\right).$$

### 2.4. Statistical Analysis

The data were analyzed using SigmaPlot software (Systat Software, Inc., San Jose, CA, USA). To determine the color coordinate values of the biofilms produced by *A. arilaitensis* affected by initial pH, additional NaCl concentration, and deacidifying yeasts, one-way analysis of variance (one-way ANOVA) was performed in order to compare mean values among the whole data for each factor, while multivariate analysis of variance (MANOVA) was applied when determining the combined effects of controlled factors on the color of *A. arilaitensis* Po102 biofilms. The differences of considered variable were estimated by Tukey and Holm–Sidak tests according to an α risk of 5% and 1% for one-way ANOVA and MANOVA, respectively.

## 3. Results

### 3.1. Diversity of Coloration by Arthrobacter arilaitensis Po102 on Different Deacidified Cheese-Based Media

After 28 days of simultaneous incubation at 12 °C under light conditions, the cheese-based (curd) solid media inoculated with *A. arilaitensis* Po102 were measured for color attributes using spectrocolorimetry in the CIE L*a*b* color system. Among the three types of media, depending on the method of deacidification, i.e., chemical deacidification by NaOH (CM_NaOH_), biological deacidification by the yeast strain *D. hansenii* 304 (CM_Dh304_), and biological deacidification by the yeast strain *K. marxianus* 44 (CM_Km44_), the coloration of the cultures on CM_NaOH_ showed hue alignments closer to the 90°, with a large number of the a* negative value, expanding from −2.50 to 3.30, while the b* value varied between 6.00 and 29.30 (data not shown). Most of cultures on CM_NaOH_ displayed cream or pale colors. By contrast, those on CM_Dh304_ and CM_Km44_ presented yellow colors, due to their hue alignments, which were set onto the color plane between 80.50 and 105.85°. The results indicated a diversity of colors among these three media, particularly between CM_NaOH_ and CM_Dh304_, whose hues were statistically significantly different (*p* < 0.05).

**Figure 1:**
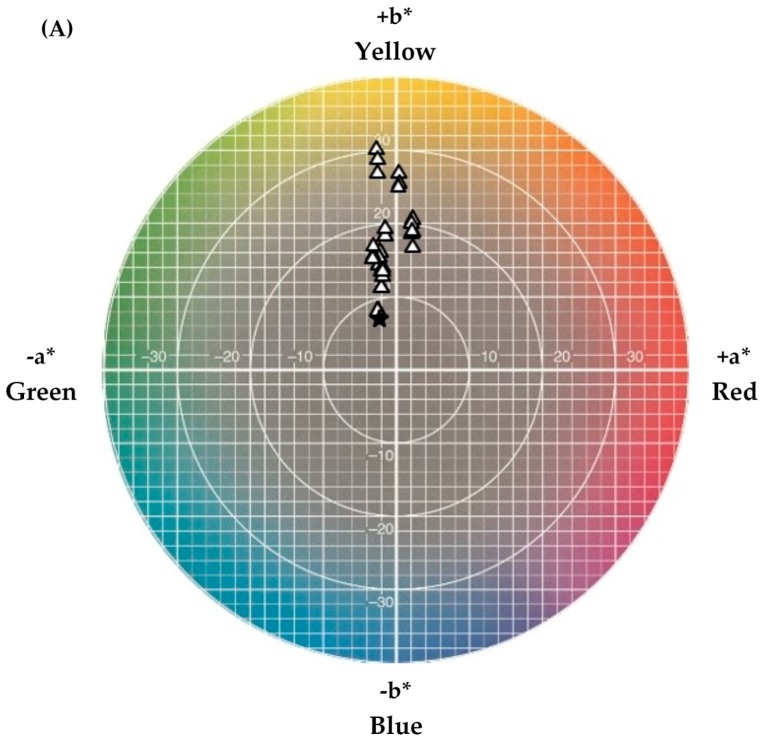

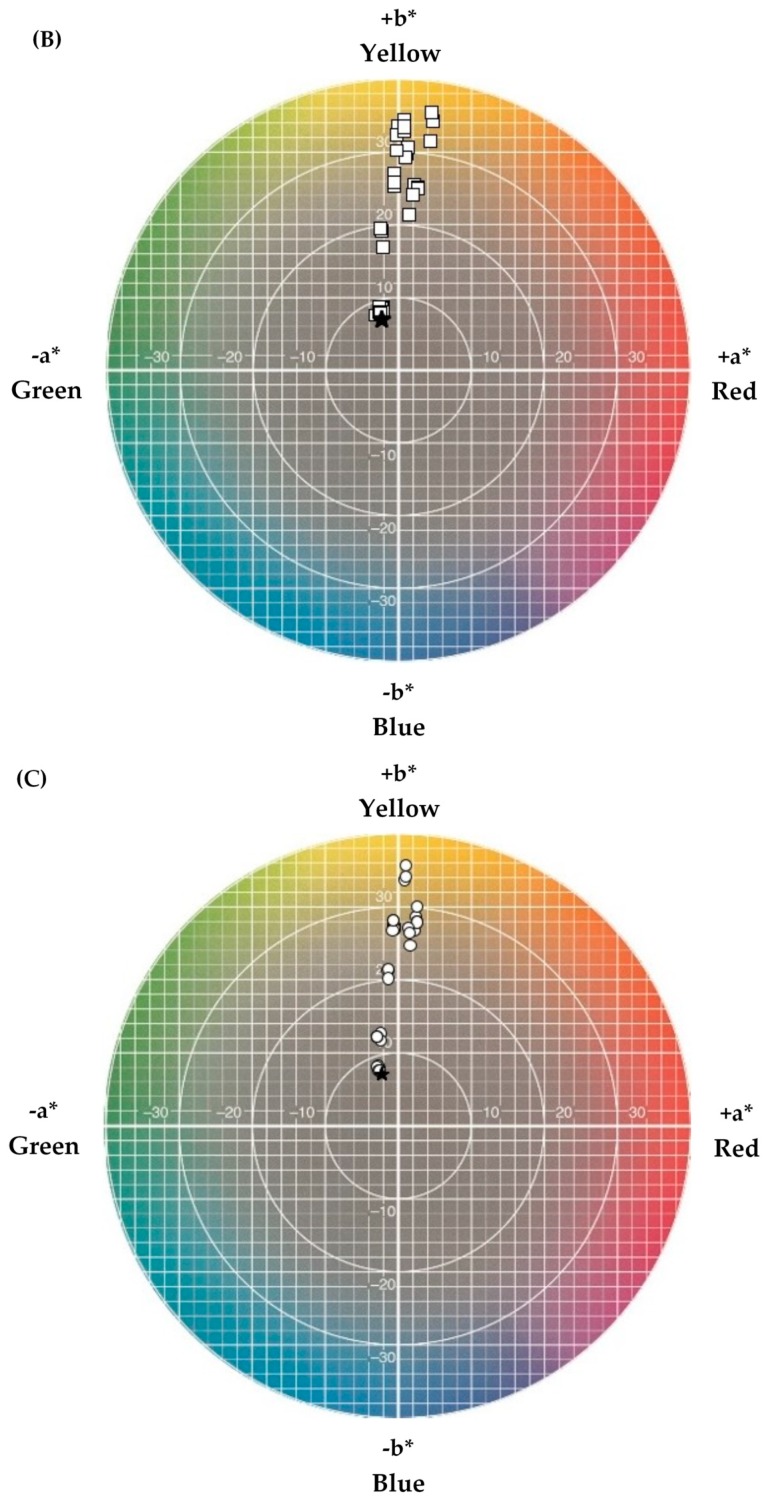
The color positions in the Compagnie Internationale de l’Eclairage (CIE) L*a*b* colorimetric system of *Arthrobacter arilaitensis* Po102 cultures cultivated on 3 kinds of deacidified cheese-based solid media. ∆ = color on CM_NaOH_ (**A**); □ = color on CM_Dh304_ (**B**); ○ = color on CM_Km44_ (**C**); and 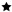 = color of blank agar (without inoculating with *A. arilaitensis* Po102).

The color position details of biofilms produced by *A. arilaitensis* Po102 on CM_NaOH_, CM_Dh304_, and CM_Km44_, coordinating with different initial pH and NaCl concentrations, are shown in Figure 1. The color projected onto a (a*, b*) plane, among the same deacidified media, showed a significant difference between the cultures in media with initial pH at 5.8, and those of 7.0 and 7.5 (*p* < 0.05), while there was no significant difference between pH 7.0 and 7.5 (*p* > 0.05). A narrow range of color coordinate values was obvious, and demonstrated at all concentrations of NaCl. However, the colors among the entire set with an initial pH 7.0 was wider distributed on (a*, b*) than those with other pH values in all types of media.

### 3.2. The Effects of pH, NaCl, and Deacidification on the Coloration of Arthrobacter arilaitensis Po102

#### 3.2.1. Changes in Color Development of *Arthrobacter arilaitensis* Po102

The color characteristics in terms of hue angle *(h*°) and chroma (saturation, C*), under the influences of pH, NaCl, and deacidification, are shown in Table 1. According to the analysis of variance, there was a significant interaction between these variables on hue and chroma of the cultures of *A. arilaitensis* Po102 (*p* < 0.001). The hue angles extended from 80.56 to 105.74, with a large number of data showing responses close to *h*° value obtained from the blank agar, which corresponded to a cream or pale color (poorly colored). Among the same type of media, *h*° values decreased when cultivated on agars with initiated pH values of 7.0 and 7.5, leading to displaying more colored cultures than those with initial pH at 5.8, especially on media deacidified by both yeast strains, *D. hansenii* 304 and *K. marxianus* 44, which presented intense yellows at all levels of NaCl concentration. When 8% (w/v) NaCl was added into these media in coordination with initial pH at 5.8, the *A. arilaitensis* strain was likely not capable of forming color. By contrast, in the absence of NaCl, the cultures of all types of media showed yellow colors, with *h*° values of 87.04, 80.85, and 82.20° for CM_NaOH_, CM_Dh304_, and CM_Km44_, respectively. 

Similar to hue, pH variations, NaCl concentration, and deacidification affected the saturation, C*, of *A. arilaitensis* Po102 biofilms. The C* responses of these biofilms widely ranged from 6.56 to 38.54, whereby a large diversity of different groups obtained significantly differing results (*p* < 0.001). At pH value of 5.8, for all 3 kinds of media, the C* values of biofilms were higher at 0% of NaCl while, at other NaCl concentrations, the saturation was much lower. However, the lowest saturations of color were measured for all types of media, at all pH levels, for 8% (w/v) of additional NaCl content.

**Figure 2:**
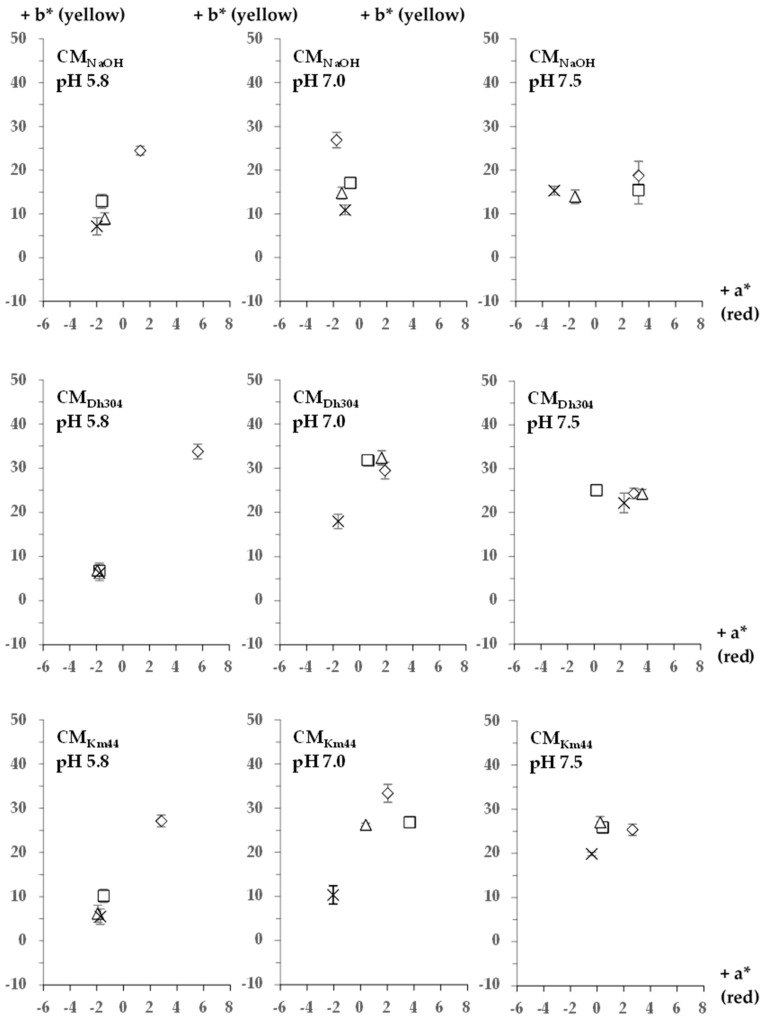
Detailed positions of *Arthrobacter arilaitensis* Po102 cultures when projected in the CIE L*a*b* colorimetric scale (bars indicate standard deviations) after day (d) 28 incubation. The concentration of additional NaCl (w/v) is presented in colors as follows: 0% (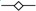), 2% (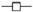), 4% (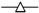), and 8% (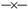).

The color attributes of *A. arilaitensis* Po102 cultures versus time were observed during the cultivation period on day (d) 7, 14, 21, and 28. Detailed positions of cultures of the *A. arilaitensis* strain, when projected in the CIE L*a*b* colorimetric scale after d 28 incubation, are shown in Figure 2. Among the same category, i.e., initial pH and NaCl concentration, the overall changes during the cultivation on CM_Dh304_ and CM_Km44_ appeared to be dependent on these variables. With the exception of the set at 0% NaCl, the color coordinate values obtained from all types of media at pH value 5.8, evolved very slightly throughout the cultivation period.

**Figure 3:**
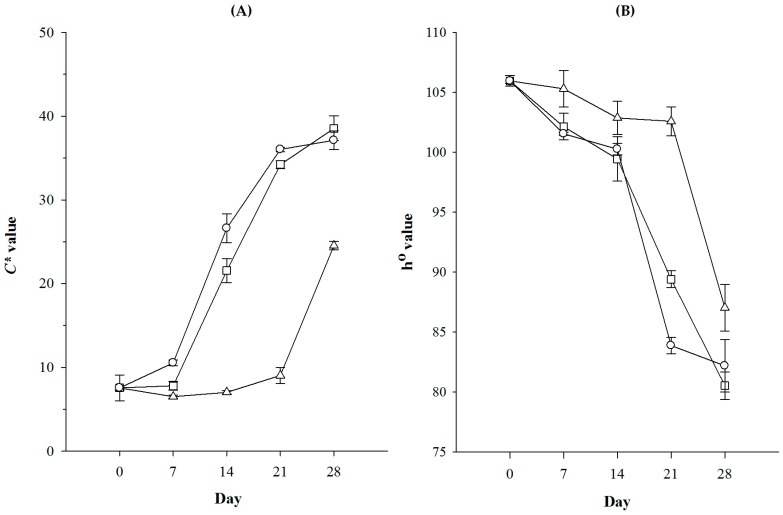
Changes in color saturations (**A**) and hues (**B**) of *Arthrobacter arilaitensis* Po102 cultures during growth on different deacidified cheese-based solid media at initial pH 5.8 without adding NaCl. ∆ = CM_NaOH_, □ = CM_Dh304_, and ○ = CM_Km44_.

On the other hand, when considering the cultures on these media with initial pH value at 5.8 without addition of NaCl, the development of color saturation of CM_Dh304_ and CM_Km44_ were similar. There were, however, strong differences between these two media and CM_NaOH_. The difference of C* values between CM_NaOH_ and the 2 others was obvious after d 7, and this remained until d 28 (Figure 3). In contrast to CM_Dh304_ and CM_Km44_, the C* responses of the *A. arilaitensis* strain cultures on CM_NaOH_ slightly increased from the beginning until d 21, whose values were not significantly different from those of the blank agar (*p* > 0.05). Then, in between d 21 and d 28, the changes in their saturation augmented rapidly, ranging from 8.99 to 24.53. Unlike the color saturation, the hue angles of the bacterial cultures were decreased throughout the incubation period. The *h*° was slightly changed during 14 days on CM_Dh304_ and CM_Km44_, and 21 days on CM_NaOH_ and, as is visible in the results, reduced at the end of cultivation period.

**Figure 4:**
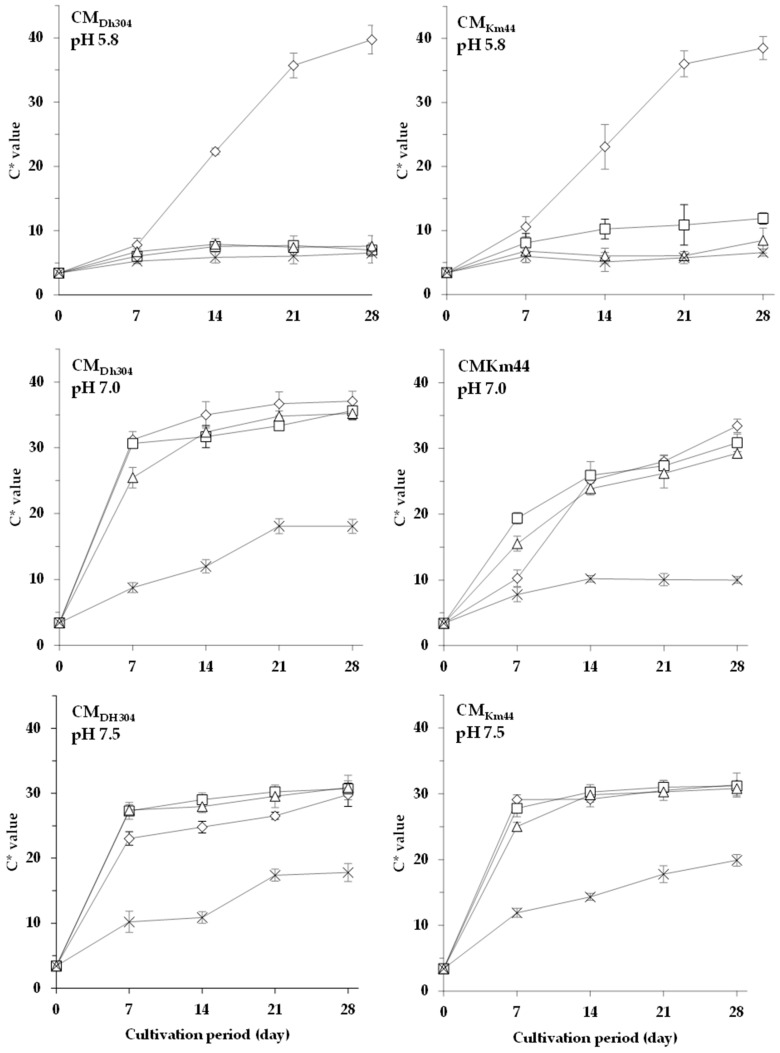
Changes in saturation C* of *Arthrobacter arilaitensis* Po102 cultures during cultivation on CM_Dh304_ and CM_Km44_ at the initial pH 5.8, 7.0, and 7.5. Culture conditions in various amounts of NaCl: 0% (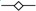), 2% (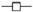), 4% (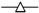), and 8% (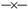).

However, the changes in these responses were different from the cultures found on the biologically deacidified media coordinated with initial pH values higher than 5.8. The color development of *A. arilaitensis* Po102 on CM_Dh304_ with initial pH values of 7.0 and 7.5, and on CM_Km44_ at pH 7.5, containing between 0–4% NaCl (w/v), increased extensively in the first 7 days of cultivation. The color of these cultures was changed from white-cream to yellow. Afterwards, from d 7 to d 28, the increase of saturation and hue slowed down. Although almost all color development patterns of the *A. arilaitensis* strain on CM_Km44_ were similar to those of CM_Dh304_, the different forms of color evolution were apparently visible when coordinated with initial pH at 7.0. The changes of saturation C* provided by this strain on the two biologically deacidified media, in relation to initial pH and concentration of NaCl, versus time, are shown in Figure 4. L* values of the *A. arilaitensis* cultures on d 28 varied between 60.34 and 78.52, over all data observed in the present study (data not shown). Regardless of NaCl concentration, the highest lightness of the cultures from among all media was found at pH value of 7.0. Like the changes in color saturation, L* values appeared to depend on the controlled factors; in addition, the pattern of the change in lightness, in terms of period, was similar to those of chroma changes in CM_Dh304_ and CM_Km44_ conditions with initial pH values of 7.0 and 7.5.

**Figure 5:**
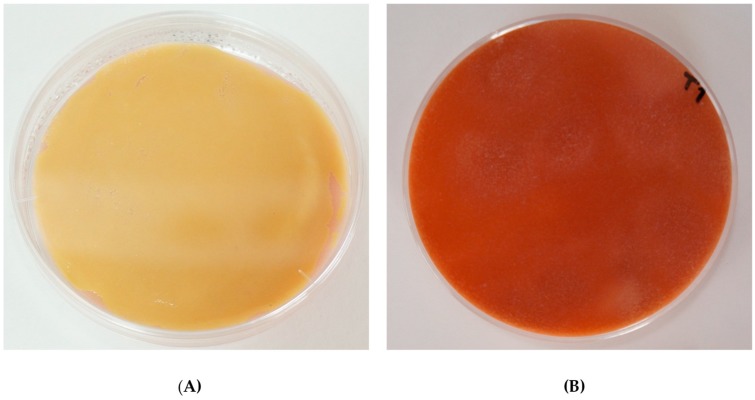
Coloration by *Arthrobacter arilaitensis* Po102 on CM_Dh304_ at the initial pH 7.0 without NaCl, as examples for pink coloration observed on the cultures in this study. (**A**) Front view; (**B**) back view.

Apart from the yellow pigments synthesized by *A. arilaitensis* Po102, it was likely that this strain also produced other pigment(s) diffusing inside the cultures. Indeed, a large number of cheese curd-based agar plates inoculated with this bacterial strain displayed a pink/red-brown color (Figure 5). The pink appeared slightly on certain plates of cultures on the three kinds of deacidified media during the first week of the incubation time. The *A. arilaitensis* strain was found to produce pink pigments on CM_NaOH_ and CM_Km44_ with the initial pH value of 7.5, while the pink pigmentation exhibited on CMDh304 when the initial pH of the media were 7.0 and 7.5. However, only with 0–2% NaCl in CM_NaOH_ and less than 8% NaCl in CM_Dh304_ and CM_Km44_, did the cultures display the pink coloration. By contrast, the other cultures that were treated differently, showed the red-brown color on d 21 and 28, respectively, including (i) CM_Dh304_ and CM_Km44_, in combination with initial pH value of 7.0 and 7.5, with added 8% (w/v) NaCl; and (ii) all types of deacidified media at pH value of 5.8, without additional NaCl.

A short experiment was then performed to determine these pigments, in order to obtain the first information for further investigation on the role of pink pigmentation by *A. arilaitensis* in smear-ripened cheeses. The results showed that the pink pigments excreted by *A. arilaitensis* Po102 were water-soluble, and the extracts exhibited fine structure with wavelengths of maximum absorption at 394, 497, 530, 567, and 620 nm; in addition, the extract fluoresced under UV light. These basic occurrences were similar to data obtained from the red-brown pigments produced by *A. nicotianae*, which were not precisely identified in the study of Bockelmann et al. [26]. Based on the review of pigments produced by the genus *Arthrobacter* [27], most bacteria in this genus could produce pigments in a broad range of hues, e.g., orange and yellow (riboflavin, carotenoids), blue and green (indigoidine, indochrome, and derived salts), and red (porphyrins, carotenoids). However, in comparison with the general porphyrin characteristics, including absorption spectra and fluorescence property, as well as the recent information reported by Cleary et al. [28], the pink/red-brown observed in the present study would probably be pigments in a porphyrin cluster.

#### 3.2.2. Changes in pH of *Arthrobacter arilaitensis* Po102 Cultures

Since the color development appeared not only on the surface but, also, inside the media inoculated with *A. arilaitensis* Po102, the cultures were blended and were then measured for their pH. When considering the whole experimental pH data of the present study, all controlled variables seemed to potentially influence the changes of pH values of the cultures. Differences in changes of pH values appeared between each type of deacidified media, especially between the chemical deacidified medium and the biologically deacidified media by yeast strains. The comparison of changes in pH of the cultures, categorized by the biological deacidification methods, could help distinguish some configuration of these changes. With an initial pH value of 5.8, two major patterns among CM_Dh304_ and CM_Km44_ were categorized, i.e., (i) slightly increased or decreased throughout the entire cultivation period, and (ii) slightly changed during the first 14 days and, then, highly increased afterward.

**Figure 6:**
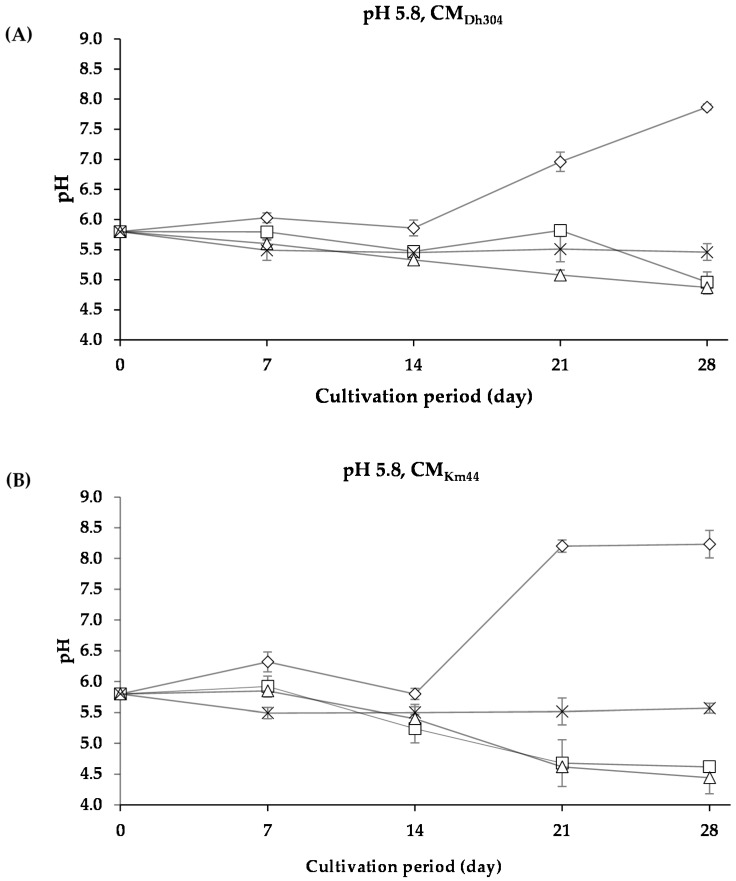
The changes in pH of *Arthrobacter arilaitensis* Po102 cultures during cultivation on CM_DH304_ and CM_Km44_ with initial pH value of 5.8. (**A**) Changes in pH of the cultures on CM_Dh304_; (**B**) on CM_Km44_. Culture conditions in various amounts of NaCl: 0% (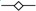), 2% (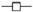), 4% (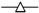), and 8% (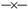).

**Figure 7:**
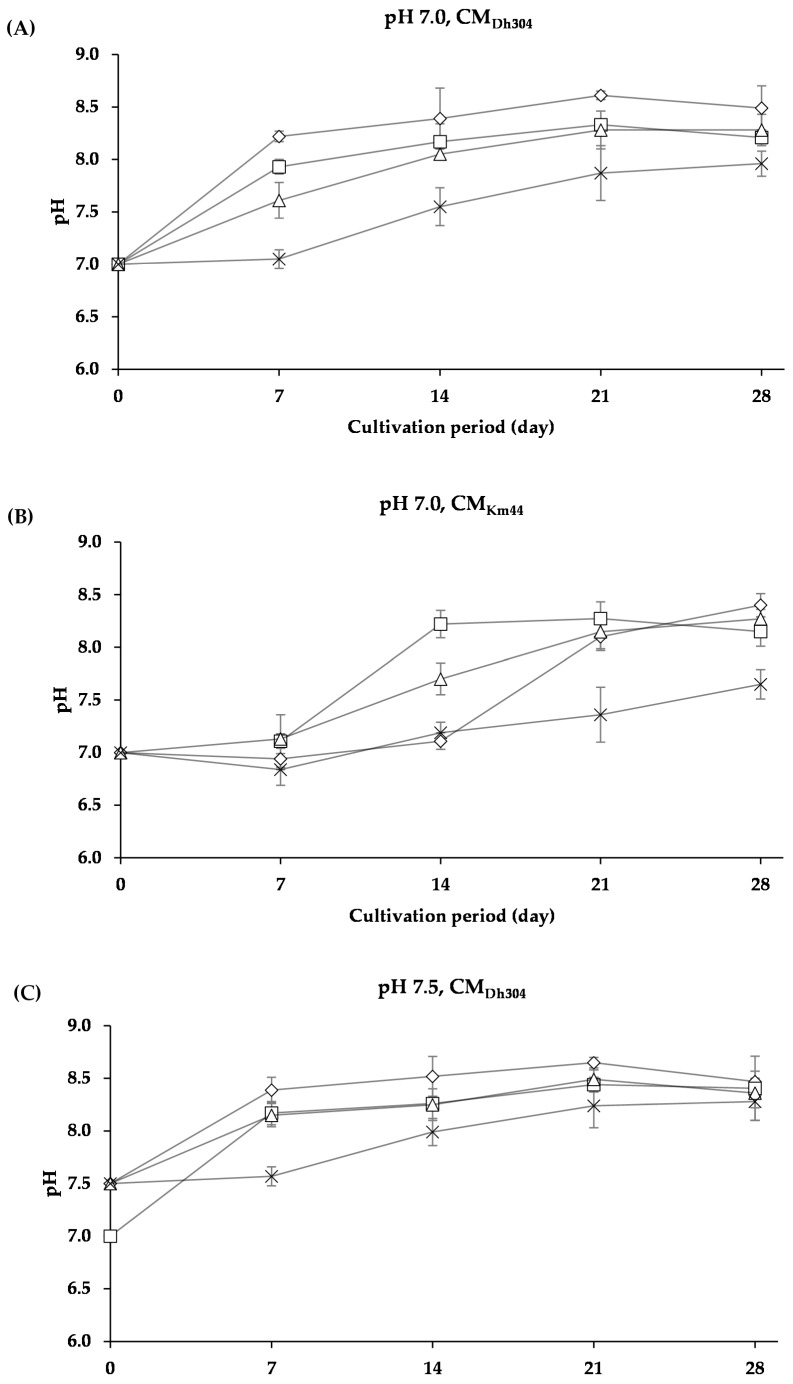

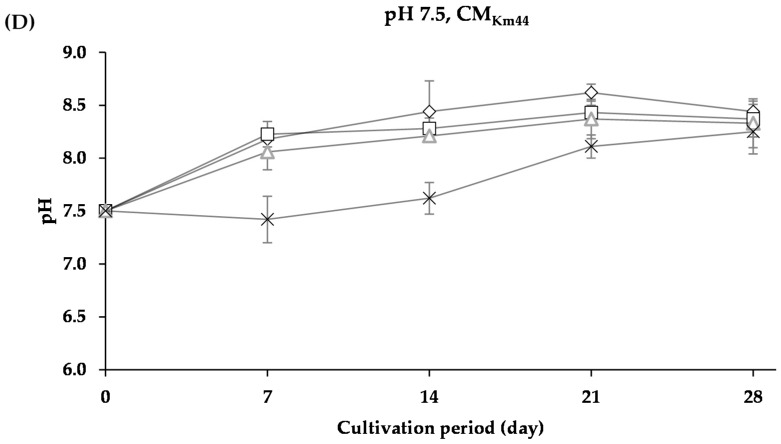
The changes in pH of *Arthrobacter arilaitensis* Po102 cultures during cultivation on CM_DH304_ and CM_Km44_ with initial pH value of 7.0 and 7.5 (**A**) Changes in pH of the cultures on CM_Dh304_ with initial pH value of 7.0; (**B**) on CM_Km44_ with initial pH value of 7.0; (**C**) on CM_Dh304_ with initial pH value of 7.5; (**D**) on CM_Km44_ with initial pH value of 7.5. Culture conditions in various amounts of NaCl: 0% (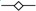), 2% (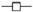), 4% (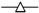), and 8% (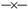).

As shown in Figure 6, pH of the cultures of *A. arilaitensis* Po102 changed slightly when the bacterial strain grew on the media containing NaCl concentrations, whereas the pH changes were manifest when grown on media without NaCl. However, when the initial pH value was higher than 5.8, patterns of the changes in pH of *A. arilaitensis* Po102 cultures were dissimilar. Two different patterns were clearly observable on both CM_Dh304_ and CM_Km44_, with initial pH values of 7.0 and 7.5, i.e., (i) pH augmented rapidly during the first 7 days, and after that, slowly, and (ii) pH changed slightly during 7 or 14 days, and then increased rapidly (Figure 7).

In the present study, pH changed during the growth, and seemed to be correlated with the development of pink color by *A. arilaitensis* Po102 on the cheese-based solid media studied. Regardless of the concentration of additional NaCl and the method used for deacidification, all of experimental agars inoculated with this bacterial strain would intensely present a pink color when their pH was increased to higher than 7.5 during the cultivation (data not shown).

## 4. Discussion

During the last few years, *A. arilaitensis* has been identified as one important bacterial strain responsible for the coloration of smear-ripened cheeses; however, to our knowledge, only the study by Leclercq-Perlat et al. [29] investigated the coloring capacity of this species on a variety of deacidified media prepared from the curd of several ripened cheeses, including Epoisses, Munster, Livarot, and Reblochon. On the other hand, a number of previous studies had focused on *B. linens* for its involvement in the cheese coloration in relation with various factors, such as pH, salt concentration, humidity, light, temperature, and yeast used for deacidification, as well as on the interactions between the *B. linens* strain and other microorganisms isolated from ripened cheeses. The present study is the first investigation which concentrated on the pigmentation of *A. arilaitensis* under the influence of initial pH, NaCl content, and deacidifying yeasts.

According to the data obtained using spectrocolorimetry in the CIE L*a*b* color system, *A. arilaitensis* Po102 provided diverse color characteristics on three types of deacidified media. However, most of the biofilms on CM_NaOH_ displayed *h*° values in the greenish-yellow range, while hues of CM_Dh302_ and CM_Km44_ were set in the reddish-yellow range. The *h*° values on CM_NaOH_ in the present study were similar to the previous study investigating the pigments produced by a variety of the *A. arilaitensis* strains. They showed the color characteristic of yellow *A. arilaitensis* strains with hues varying from 92.61 to 98.57 [9]. This could be explained by the resemblance of the media used for the 2 studies, as both of them had an initial pH at 7.0, adjusted by using the chemical reagent NaOH. Besides, this bacterial species was reported to be sensitive to the type of media used for its cultivation, as its color attributes were distinctly different when grown on media prepared from various cheese curds [29].

All controlled variables i.e., initial pH, NaCl concentration, and deacidifying yeasts, appeared to affect the pigments produced by *A. arilaitensis* Po102. When the pH value was initiated at 5.8, this bacterial strain was likely to notably produce pigments in the absence of NaCl. As established by the plate counting technique, the viable cell concentration of the *A. arilaitensis* strain was significantly higher without NaCl compared to cultures grown on media containing 2–8% (w/v) NaCl (data not shown). This incidence could imply that the color intensity of biofilms was directly proportional to the number of bacterial cells. As a result, it also potentially pointed out a direct effect of initial pH on the growth of this *A. arilaitensis* strain. These results were consistent with the previous studies on the pigmentation of *B. linens*, which reported a similar relation between pH, viable cell concentration, and color intensity [21,29].

A comparison of the color attributes provided by *A. arilaitensis* Po102 on either CM_Dh304_ or CM_Km44_, at initial pH values of both 7.0 and 7.5, apparently showed differences between the cultures containing 8% (w/v) NaCl and the other ones. Meanwhile, the color attributes were quite similar among the cultures on media with 0–4% (w/v) NaCl added. In the same way, these differences seemed to depend on the intensity of viable cells as the number of viable *A. arilaitensis* Po102 on plate count agar was significantly lower when grown on media containing 8% (w/v) NaCl compared to agars with other concentrations (data not shown).

When considering the color of *A. arilaitensis* Po102 cultures on CM_Dh304_ and CM_Km44_ at initial pH values higher than 5.8, with 0–4% (w/v) NaCl, whose conditions were probably more suitable for the growth of the *A. arilaitensis* strain in the present study, the color attributes, including a*, b*, C*, and *h*° value, varied greatly among these media. In addition, during the incubation period, the changes in color and pH were diversified. These results could refer to the relation between the yeast strains used for the deacidification, and the pigment production of this bacterial strain. For the media prepared by biological deacidification, yeast cells were eliminated by pasteurization when the pH of the media reached the value required. For this reason, the pigmentation of *A. arilaitensis* Po102 was possibly impacted by the difference in biochemical composition of CM_Dh304_ and CM_Km44_. Leclercq-Perlat et al. [17] reported that *K. marxiamus* required more lactose and lactate than *D. hansenii* for their growth during the deacidification; therefore, the ripening bacteria probably did not have sufficient lactate to produce energy for their growth. This resulted in a variation of the cell biomass, which directly influenced the color. Furthermore, the ability to produce pigments of *A. arilaitensis* appeared to depend on the type of amino acids and caseinate present in the milk media [30]. According to Roostita and Fleet [30], *D. hansenii* was able to produce several free amino acids (predominantly proline, alanine, glycine, arginine, and glutamic acid) and free fatty acid, during its growth in milk. This probably affected the growth and, thus, pigment production of the ripening bacteria *A. arilaitensis* Po102.

In the present study, the development of pink/red-brown colors on all types of deacidified media was evident in cultures produced by *A. arilaitensis* Po102, as shown in Figure 5. The initial pH of cheese-based solid media seemed to implicate the pink coloration of this bacterium, as there was no development of pink color when the pH was acidic (pH 5.8). Unlike the initial pH, NaCl appeared not to directly affect the pink pigmentation of the *A. arilaitensis* strain because the red pigments were found in cultures on media which had an initial pH at 7.0 and 7.5, containing all NaCl concentrations. However, after observing the changes in pH during its growth, it could be indicated that the *A. arilaitensis* strain would produce pink pigments when the pH value of the inoculated media were higher than 7.5. Bockelmann [4] reported that a ripening bacteria, *A. nicotianae*, a yellow-pigmented bacteria isolated from German cheeses, produced red-brown pigments when co-cultivated with *D. hansenii* and other bacteria under alkaline growth conditions in a shake liquid milk model system. However, the pure culture of this strain also produced red-brown pigments in liquid growth media containing casein hydrolysate. The same author demonstrated a possibility to use this bacterial species as a ripening culture for the typical red-brown Tilsit cheese. The pink coloration, sometimes identified as pink-brown or dark-brown, has often been reported as a defect in a wide variety of smear-ripened cheeses, which influences the cheese quality, and is rejected by consumers [31,32]. Many microorganisms as primary starters and secondary cultures have been implicated in the pink coloration in cheeses; however, to our knowledge, the pink pigments produced by *A. arilaitensis* have not yet been clearly and fully identified in many experimental conditions.

## 5. Conclusions

The color of cultures provided by *A. arilaitensis* Po102 on cheese-based (curd) solid media were prepared using different deacidification methods, i.e., (i) chemical deacidification by NaOH (CM_NaOH_), (ii) biological deacidification by the yeast strain *D. hansenii* 304 (CM_Dh304_), and (iii) biological deacidification by the yeast strain *K. marxianus* 44 (CM_Km44_). In association with varied initial pH values and NaCl in varying concentrations, the cultures on all kinds of media were analyzed by spectrocolorimetry in the CIE L*a*b* color system after 28 days of cultivation at 12 °C under light conditions. According to collected data, all controlled factors appeared to affect the pigments produced by the *A. arilaitensis* strain, in terms of hue, saturation, and lightness. NaCl content in the media showed distinct inhibitory effects on the development of color by this strain when the initial pH was at 5.8. By contrast, when the initial pH of the media was higher (7.0, 7.5), only the highest concentration of NaCl (8%) had this effect. The coloring capacity of this bacterial species was generally higher when *D. hansenii* 304 was used for the deacidification, compared to *K. marxianus* 44. Based on the pH measurement, the yeast strains *D. hansenii* 304 and *K. marxianus* 44 appeared to impact, differently, a change in pH of the cultures. These changes were assumed to be associated with the occurrence of pink/red-brown color in the *A. arilaitensis* cultures, as the pink coloration manifested itself when the pH of the cultures was higher than 7.5. In general, the pink color in smear-ripened cheeses is defined as a defect. Therefore, in order to avoid this defect in the cheese production, the correlation between these factors, particularly, the change in pH during ripening, should be further observed. Different deacidifying yeast strains, mainly found in smear-ripened cheeses, would possibly be applied, and the change in pH throughout the ripening period should be critically focused on. The potential results may indicate the optimum pH that would maintain the growth and color development of *A. arilaitensis* or other pigmented bacterial strains, but not induce the red pigments. This will be useful for the cheese production to prevent economic losses from the pink discoloration in cheeses.

## Figures and Tables

**Table 1:** The *h*° values and the saturation (C*) of *Arthrobacter arilaitensis* Po102 cultures on cheese curd-based solid media deacidified by NaOH (CM_NaOH_), *Debaryomyces hansenii* 304 (CM_Dh304_), and *Kluyveromyces marxianus* 44 (CM_Km44_) after incubation at 12 °C under light conditions for 28 days.

pH	NaCl (w/v)	CM_NaOH_		CM_Dh304_		CM_Km44_	
		*h*°	C*	*h*°	C*	*h*°	C*
5.8	0%	87.04 ± 1.95 ^m^	24.53 ± 0.51 ^b^	80.53 ± 1.15 ^bc^	38.54 ± 1.48 ^a^	82.20 ± 2.20 ^b^	37.12 ± 1.12 ^a^
	2%	100.26 ± 1.02 ^a^	14.46 ± 0.13 ^cd^	104.19 ± 0.39 ^n^	7.71 ± 0.62 ^m^	98.37 ± 1.09 ^a^	13.60 ± 1.72 ^c^
	4%	102.59 ± 0.83 ^o^	9.66 ± 1.04 ^n^	104.46 ± 0.88 ^p^	7.88 ± 1.08 ^o^	104.26 ± 1.28 ^q^	6.79 ± 0.54 ^p^
	8%	105.53 ± 2.68 ^r^	7.42 ± 0.25 ^q^	105.74 ± 0.73 ^s^	6.71 ± 0.18 ^r^	105.25 ± 0.63 ^t^	6.56 ± 1.44 ^s^
7.0	0%	93.76 ± 0.76 ^u^	31.41 ± 0.69 ^d^	86.13 ± 0.72 ^de^	31.24 ± 2.23 ^t^	84.80 ± 1.67 ^d^	33.43 ± 1.17 ^u^
	2%	92.44 ± 1.43 ^i^	17.10 ± 0.25 ^v^	87.69 ± 2.13 ^v^	33.33 ± 2.31 ^w^	85.05 ± 2.31 ^w^	26.91 ± 0.14 ^e^
	4%	95.35 ± 0.28 ^j^	14.81 ± 0.34 ^x^	87.08 ± 0.13 ^g^	35.17 ± 3.28 ^y^	89.13 ± 0.13 ^gh^	29.22 ± 2.58 ^l^
	8%	95.12 ± 0.18 ^kl^	12.53 ± 0.94 ^g^	95.18 ± 0.38 ^l^	18.08 ± 2.42 ^h^	100.35 ± 1.25 ^x^	11.15 ± 1.55 ^g^
7.5	0%	80.86 ± 0.67 ^y^	25.37 ± 0.96 ^b^	83.04 ± 1.83 ^z^	26.18 ± 1.05 ^z^	83.47 ± 0.74 ^a^*	29.50 ± 1.53 ^a^*
	2%	94.46 ± 1.16 ^i^	24.17 ± 0.17 ^b^*	88.51 ± 0.37 ^ef^	30.19 ± 1.39 ^f^	88.85 ± 2.23 ^f^	29.86 ± 0.94 ^ef^
	4%	96.33 ± 0.63 ^j^	23.70 ± 2.47 ^c^*	81.56 ± 0.56 ^b^*	31.89 ± 1.35 ^k^	89.52 ± 1.39 ^h^	29.84 ± 1.85 ^kl^
	8%	99.55 ± 1.42 ^k^	22.16 ± 0.55 ^d^*	83.22 ± 2.05 ^c^*	17.46 ± 1.74 ^hi^	91.17 ± 0.33 ^d^*	19.87 ± 0.97 ^ij^

Values in the same category with a common superscript letter do not significantly differ (*p* > 0.001).
